# Benefits of a non-traditional science communication and internship experience based on research from the National Science Foundation Research Traineeship at a Research Intensive University

**DOI:** 10.1371/journal.pone.0320372

**Published:** 2025-04-22

**Authors:** Sukanya Dasgupta, Tayler Schillerberg, Haven Cashwell, Chandana Mitra, Karen S. McNeal

**Affiliations:** 1 Department of Geosciences, Auburn University, Auburn, Alabama, United States of America; 2 Department of Crop, Soil, and Environmental Sciences, Auburn University, Auburn, Alabama, United States of America; 3 USDA Midwest Climate Hub, Ames, Iowa, United States of America; 4 State Climate Office, North Carolina State University, Raleigh, North Carolina, United States of America; Ataturk University, Faculty of Pharmacy, TÜRKIYE

## Abstract

Effective science communication and stakeholder engagement are crucial skills for climate scientists, yet formal training in these areas remains limited in graduate education. The National Science Foundation Research Traineeship (NRT) at Auburn University (AU) addresses this gap through an innovative program combining science communication training with co-production approaches to enhance climate resiliency of built, natural, and social systems within the Southeastern United States (US). This paper evaluates the effectiveness of two novel graduate-level courses: one focused on science communication for non-technical audiences and another combining co-production methods with practical internship experience. Our research employed a mixed-methods approach, including a comprehensive analysis of course catalogs from 146 research-intensive universities and qualitative assessment of student experiences through surveys and descriptive exemplars. Analysis revealed that AU’s NRT program is unique among peer institutions in offering both specialized science communication training and co-production internship opportunities to graduate students across departments. Survey data from 11 program participants and detailed case studies of three program graduates demonstrated significant professional development benefits. Key outcomes included enhanced stakeholder engagement capabilities, improved science communication skills, and better preparation for both academic and non-academic careers. These findings suggest that integrating structured science communication training with hands-on co-production experience provides valuable preparation for climate scientists. The success of AU’s program model indicates that similar curriculum structures could benefit graduate programs nationwide, particularly in preparing students to effectively communicate complex scientific concepts to diverse audiences and engage with stakeholders in climate resilience efforts.

## Introduction

It has become more common in recent years to encourage academics in Science, Technology, Engineering, and Math (STEM) to conduct applied research usable to stakeholders and society [1–3]. Unfortunately, STEM graduate student training rarely includes opportunities to work with outside stakeholders, and even less often are students trained in working with such stakeholders or communicating their science to non-technical audiences [4]. In this paper, we analyze research-intensive (R1) institutions in the United States (US), and their official offerings for courses that relate to either science communication (emphasizing communication to non-technical audiences) or co-production internship experiences (collaboratively based with outside stakeholders) for graduate students through intensive scraping of all current course catalogs, syllabi, and bulletins [5]. Our data illustrates the dearth of graduate courses aimed to promote science communication or stakeholder partnerships and engagement; thus, the limited opportunities graduate students have nationwide to develop these skills.

## Objectives

The National Science Foundation Research Traineeship (NRT) program aims to transform graduate education through innovative graduate training programs across all STEM fields with projects that focus on interdisciplinary research topics of high national priority to implement a range of educational training that promotes skill development including technical, research ethics, communication, entrepreneurship, and cultural competency among others and expose students to a range of STEM careers where institutionalization of successful project elements beyond the project lifetime is expected [6]. The objectives of this paper are to analyze the qualitative open-ended response survey data describing perspectives of Auburn University’s (AU) NRT students who have completed two NRT developed courses and provide perspectives of their experiences with the internship. Alongside this, a comprehensive analysis of the nation’s R1 institutions for parallel existing programs was completed to better understand the availability of such courses in graduate education. This research aims to illustrate the dearth of graduate-level science communication and co-production courses offered at R1 Universities across the US and illustrate AU’s NRT courses as exemplars that can meet this need in graduate education across the country.

## Background

### Teaching and learning in STEM graduate studies

Traditional science graduate programs consist of certificates, master's degrees, doctoral degrees, or some combination of these. There are usually a required number of courses that must be completed in the chosen discipline, with the remaining courses being elective or supplementary courses and research hours. These courses are predominately taught through lecture and examination-based approaches, as these approaches have been the traditional science pathway learned and adopted by those who go into teaching [7]. Upon completion of graduate degrees, those who remain in academia often continue implementing these outdated methods in their own teaching [8–10].

Traditional approaches to lecturing and science communication, which often involve one-way dissemination of information, create limitations and are not as effective as active learning methods which are becoming more common in today’s dynamic communication landscape. First, this traditional format lacks interactivity, making it challenging for students to engage with and retain complex scientific concepts. According to a study by Freeman et al. [11], active learning methods outperform traditional lectures in terms of student performance and engagement which emphasizes the need for more interactive approaches. Second, the digital age has revolutionized information access as audiences can explore scientific topics online, often with multimedia and interactive content. This shift necessitates science communicators to adapt and embrace digital media and communication tools. Jensen & Buckley [12] suggest integrating digital technology can enhance science communication, making it more engaging and accessible.

Active learning engages the audience in a two-way conversation, fostering better understanding and retention of scientific concepts [11]. Previous research found that active learning promotes deeper comprehension by encouraging learners to construct their own knowledge [13] and improves problem-solving skills and critical thinking [14]. In an era of information overload, active learning not only captures attention but also empowers audiences to become active participants in the science communication process. Despite the overwhelming literature that supports active learning approaches in undergraduate classrooms, research in graduate education is much more limited. Regardless, graduate students report insufficient exposure to active learning and express the desire for more active learning in the graduate-level courses they take [15].

A traditional science internship is a hands-on educational experience where students or early-career professionals work under the guidance of experienced scientists to gain practical skills and knowledge in a specific scientific field [16]. These internships typically involve laboratory or field research, data collection, analysis, hands on training, and collaboration with research teams spanning several weeks or months. They provide invaluable exposure to industry practices, enhance a participant’s resume, aid in professional development, and can lead to future employment in STEM fields, fostering career development [17–20]. In this study, traditional internships are not implemented, rather a non-traditional research internship approach is utilized where students are partnered with external organizations or agencies to conduct co-produced research that is mutually beneficial to both the student (e.g., part of their dissertation) and the stakeholder (e.g., actionable science) through the use of structured decision making processes and effective science communication..

### Co-production, structured decision making (SDM) & science communication

Co-production is “the collaborative process of bringing a plurality of knowledge sources and types together to address a defined problem and build an integrated or system-oriented understanding of the problem” [21]. The co-production framework bridges the connection between science makers and science users to enable better-produced science products and tools to be aidful and used by decision-makers [22,23]. For project success, it is essential to have genuine relationships from the beginning of the project that continue throughout with regular meetings [24] and focus on the production of the product or activities [3]. Current gaps in the co-production framework include power instability, institutional and professional barriers to funding and resources, and limited stakeholder representation [3]. Compared to traditional science approaches, the co-production approach is very goal-oriented, allowing both the researcher and stakeholder partner to feel more empowered with their work. Co-production has a more integrated process, addressing stakeholder concerns and assisting with the overall acceptance of the final product. Many studies show that co-produced research, in a copious number of fields, has produced note-worthy end products that have been helpful for all teams collaborating [3,23,25–27]. The major benefits also include shared decision-making, shared creation and design, and full focus on the end goal, which could be an action or a product [3].

Structured Decision Making (SDM), on the other hand, is a formalized approach that aids in making complex decisions based on science, values, and trade-offs. Keeney and Raiffa’s book, “Decisions with Multiple Objectives: Preferences and Value Trade-Offs” provides a foundational framework for SDM [28]. By defining clear objectives, choosing appropriate communication channels, tailoring content to the audience's level of understanding, addressing potential misconceptions, and continually evaluating the strategy, science communicators can effectively convey complex ideas to diverse audiences through SDM techniques [29–33]. Under the SDM approach, a problem is decomposed into five key elements: (1) problem framing and characterizing the scope and context of the decision, (2) defining an objective where key uncertainties are identified, characterized, and incorporated into the process, (3) identifying alternatives or actions that have the potential to lead toward the objective(s), (4) identifying consequences or predicting how a system will respond to decisions, and (5) establishing a means of optimizing the objectives [34,35]. Both co-production and SDM are invaluable tools as they ensure that scientific knowledge is not only accessible but also actively utilized in decision-making processes, ultimately contributing to more sustainable and inclusive solutions for complex environmental challenges.

Science communication is the practice of conveying scientific knowledge and discoveries to various audiences with different knowledge levels, making complex concepts accessible and engaging [36]. It plays a crucial role in bridging the gap between scientists and the general public, promoting understanding and appreciation of science. Effective science communication serves several vital purposes, such as public understanding, trust and credibility, policy influence, and inspiring future scientists [37]. It helps audiences comprehend scientific advancements, fostering a more scientifically literate society [38], and builds trust between the scientific community and the public, which is essential for public support and informed decision-making [39].

### Graduate science communication and STEM studio Internship courses at AU

One unique feature of AU’s NRT program is the coursework offered with the traineeship: Science Communication and STEM Studio. For trainees, this coursework is required doctoral students must take both courses, while master students are only required to take the science communication course (due to time to degree constraints); however, any graduate student within AU may take either of the two courses. The Science Communication course incorporates an active learning approach where each class includes a combination of lectures (either from an instructor or student lead) with active learning activities. The course begins with an introduction to science communication, emphasizing the main goal of communicating research to a range of non-technical audiences and tailoring their messaging to their intended audience by using appropriate language and presentation strategies. The primary course text includes “Don’t Be Such a Scientist: Talking Substance in an Age of Style” by Randy Olson [40]. As the course continues, students present on various course designated topics using the provided class resources and literature and are provided opportunities to interact with guest speakers from a range of science communication expertise (e.g., journalism, theater, outreach, extension, non-traditional academic/government positions, non-profit, etc.) to demonstrate the breadth of opportunities that science communication is utilized within as well as provide experiences from experts in diverse fields working in this area. Throughout the course, various assignments allow for different forms of science communication to be practiced: an online blog post, a 3-minute video about their research (with the support from AU’s library media services), and an outreach event that is held in conjunction with Earth Day on AU’s campus. These activities allow students to incorporate the theory and best practices learned in class into real-world science communication activities where they can practice and receive feedback to continue to improve their communication skills both inside and outside the classroom.

The second course developed through the NRT program, called “STEM Studio,” aims to create a connection between a graduate student and a stakeholder partner to facilitate collaboration between the two independent entities to develop and complete the non-traditional research internship. Before the start of the semester, students in STEM Studio were introduced to SDM through a workshop led by an expert in the field so students could be immersed in SDM thinking from the beginning of the course. This workshop expanded on different techniques within SDM and highlighted components of co-production as both concepts were crucial to understanding and gaining the most out of the identified internships. During the course, each student led one or more discussions around an SDM or co-production-focused research paper, which helped students understand these processes in research settings and prepare for scientific explanations around complex research to stakeholders. Another important component of this course is that a potential stakeholder was identified for each student to conduct an internship with. The internship project was brainstormed during the STEM Studio course, with students submitting a proposal of their co-produced research plan at the end of the semester and the internship was then completed the semesters following the course with students providing continued structured updates to the Studio Course instructor through an on-line classroom management portal.

For this internship, stakeholders agreed to work with a graduate student on a project of shared interest that ideally could also be incorporated into the student's research. During the semester, students met with stakeholders to develop a research proposal using SDM methods. By the end, students identified an internship project with their stakeholder. Each student presented their plan and timeline for joint approval from their stakeholder and academic advisor. This innovative framework transcends conventional internship models by fostering collaborative research between students and stakeholders, resulting in actionable outcomes that directly serve stakeholder needs while advancing scientific understanding. Thus, graduate students are afforded the benefits of career exposure, skill development, science communication, and networking of a traditional internship, but are also supported to engage in a project that aligns with their research efforts and allows for their continued progression in graduate school.

### Research questions

This study examines two primary research questions derived from the program's objectives: First, what is the current landscape of similar graduate-level science communication and co-production courses across R1 institutions in the US? Second, how do AU NRT students perceive and experience the integration of science communication and co-production courses in their graduate education, as evidenced through qualitative survey responses, and how does AU’s NRT program address potential gaps in graduate education nationwide? These questions aim to illustrate both the scarcity of such graduate-level courses at R1 Universities and demonstrate how AU’s NRT courses serve as exemplars for meeting this educational need across the country.

## Methods

### R1 institution methodology

To understand if other universities have similar programs/courses to the ones offered through AU’s NRT, an evaluation of graduate-level science communication and internship courses offered at other R1 institutions was conducted with R1 being defined by the Carnegie Classification of Institutions of Higher Education [41]. Current course catalogs, bulletins, and a secondary Google search were used to search if a university offered a science communication course or STEM Studio-like course in a similar structure to the courses offered at AU. For each university on the Carnegie list, courses were searched using a keyword search within the course bulletin, and available course descriptions were analyzed using a baseline criterion included in Table 1. Science communication courses were then sorted into three classes: *No*, *Possible*, and *Yes* and STEM Studio courses were sorted into two classes: *No* and *Yes.* STEM Studio courses were only categorized into two categories due to the lack of courses and course description availability across all R1 institutions reviewed.

**Table 1:** Criteria for establishing if there was science communication and/or co-production internship courses at each R1 institution.

Criteria for a science communication course:						
	Yes	The course description mentions “communication” to a “general” or “lay” audience.				
	Possible	The course was titled “Science Communication” but failed to meet the criteria or no course descriptions were available.				
	No	No course offerings or failed to meet requirements.				
	NA	Undergraduate course listed; however, they are not included in final analysis.				
	Keywords	“Communication”, “graduate”, “science”, “science communication”, “scientific”				
Criteria for a co-production internship course:						
	Yes	The course descriptions mention “structured decision making,” “SDM,” or “co-production” with a stakeholder.				
	No	The course description does not mention “structured decision making,” “SDM,” or “co-production,” therefore the course is assumed to be a traditional internship experience.				
	Keywords	“Co-production”, “coproduction”, “graduate”, “internship”, “stakeholder”				
Example of Excel sheet heading format used to record findings:						
	University Name	Sources Used	Course Name	Department	Description	Met Criteria?

The third row contains simplified column headings used to record data.

Course selection was limited to graduate standing, meaning that courses marketed toward undergraduate students may have been noted but were not considered for the analysis. The department and college were not limited during the search thus, both STEM and non-STEM departments were included. Departments were then classified into broader subject areas to understand which disciplines offered courses that were similar to the Science Communication and STEM Studio courses offered at AU. For example, Physical Science encompasses Chemistry, Geosciences, Physics, and other Earth Science departments within our illustration of the data. Once all R1 universities were analyzed for the specific courses, they were tallied to show how many courses were offered and in which departments they were found.

### Ethics statement

The AU Institutional Review Board (IRB) determined that the study was approved as exempt under 45 CFR 46.104 and all data was collected under IRB protocol #19–278. We received written, informed consent by all research participants in this study (e.g., NRT project trainees signed printed consent forms and further indicated their agreement to participate by clicking their consent on all online surveys). No minors were included in the study.

### AU NRT survey methodology & student perspectives

To keep anonymity, all survey results, prior to the availability of the authors, were pre-processed by members of Technical Education Research Centers (TERC), a non-profit composed of math and science education and research experts, who are externally evaluating the AU NRT program. De-identified open-ended responses were then sent to the researchers for qualitative analysis.

AU’s NRT program emphasizes the importance of the student perspective within the program. Students are given the opportunity to provide feedback to external evaluators to assess the current progress of the larger NRT program through surveys and interviews. The surveys, containing similar questions, are typically taken both at the beginning and end of the academic year. For this current study, those survey responses (N = 11) pertaining to opinions on the Science Communication and STEM Studio courses were analyzed in word clouds to understand what themes were prominent that students gained and valued from these courses. The word cloud from the AU respondents represents the key terminologies used by students, as indicated through their survey responses. For evaluation purposes, open-ended responses were entered into Excel for data compilation and then, the responses were put into R to create qualitative images of the responses. The survey included 20+ questions, in multi-form response types. Two open-ended questions were selected that targeted what outcome students were looking for going into the science communication course and what they perceived to have gained. An example of the likert and open-ended survey questions is included in Appendix S1.

In addition to surveys, this paper’s graduate student authors provide perspectives of their own experiences with the non-traditional research internship as descriptive exemplars of the co-production process.

## Results

### R1 institution course results

**Fig 1:**
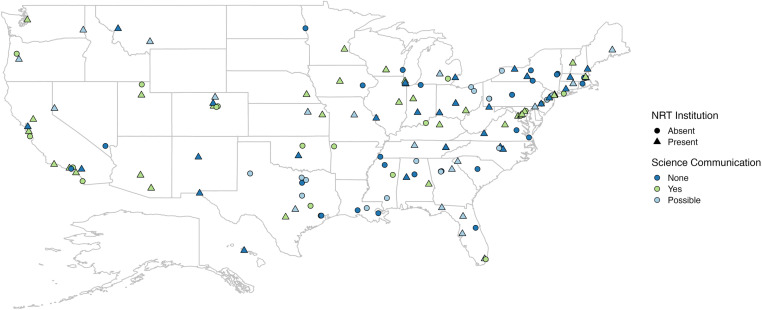
Graphical representation of R1 Institutions in the US. R1 institutions not awarded an NRT program (circles) and NRT programs awarded (triangles) are also shown. Institutions are described as having a graduate level science communication course (light green), a possible course (light blue), or no course (dark blue). For example, an institution that was awarded an NRT but does not have a science communication course would be indicated with a dark blue triangle. The map is projected using a rectangular projection.

There are 146 institutions in the US that are classified as R1 institutions [5]. These institutions are mostly concentrated on the Eastern side of the US, becoming more sporadic west of the 100^th^-degree meridian line with higher concentrations in Colorado and California (Fig 1). Because the Science Communication and STEM Studio courses were developed out of the NRT project, the NRT award status was also noted for each R1 institution. Of the R1 institutions, 55% were awarded an NRT program, which can last from five to six years. The NRT awards, with funding ranging from two to three million dollars, vary by institution, focusing on different research areas and program goals [6].

Fig 1 shows where science communication courses are found across all R1 institutions. Over half (51.3%) of the R1 institutions did not have a graduate level science communication course where the course description included communication to a general or non-academic audience. For nearly one-third (29.5%) of the R1 institutions, we were unable to find any mention of a graduate level science communication course, including any courses that focus solely on academic audiences, professional presentations, or communication. When just observing NRT-R1 institutions, at the time these data were collected, one-quarter (25.9%) did not have any form of graduate level science communication course. This one-quarter does not include various science communication workshops or seminars that institutions may offer in lieu of more formal courses.

**Fig 2:**
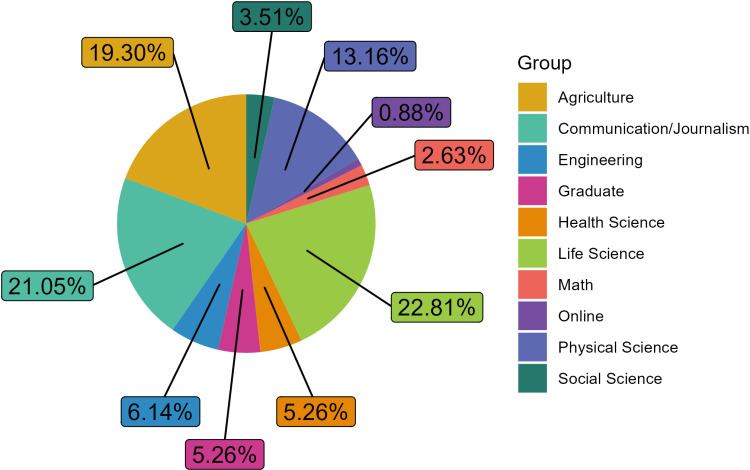
Graduate level science communication courses that met the criteria were grouped into common departmental themes. Unique classifications like “Graduate” indicate that the course was generalized and offered by the Graduate School, “Online” indicates that the course was only offered online, and the course does not appear to be housed in a specific department.

**Fig 3:**
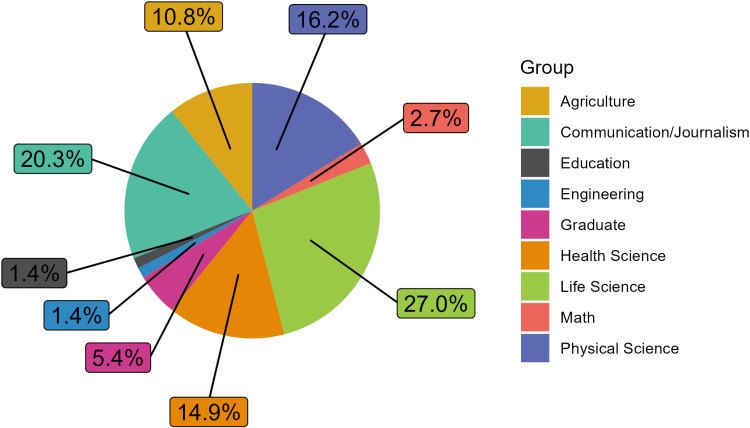
Graduate level science communication courses that did not meet the criteria were grouped into common departmental themes. Unique classifications like “Graduate” indicate that the course was generalized and offered by the Graduate School, “Online” indicates that the course was only offered online, and the course does not appear to be housed in a specific department.

Fig 2 and 3 showcase the departments in which science communication courses were housed, with Fig 2 demonstrating where the criteria “communication to a general audience” was met. The majority of science communication courses that met the criteria were from the Agriculture, Life Sciences, and Communication departments across the universities. The departments that had the least number of science communication courses were Social Science and Mathematics. In total, there were 114 graduate level science communication courses that met the criteria. The cross-listed courses were analyzed using the departmental area of study most represented. Universities with more than one science communication course that were not cross listed were analyzed individually.

Fig 3 displays the departments that housed the science communication courses that did not meet the criteria. There were 74 courses that did not meet the criteria. Similar to the science communication courses that met the criteria, there were two departments that were represented the most frequently: Life Sciences and Communication. However, the Health Sciences, Physical Sciences, and Agriculture departments were represented close behind the other departments, with a similar number of science communication courses not meeting the criteria.

**Fig 4:**
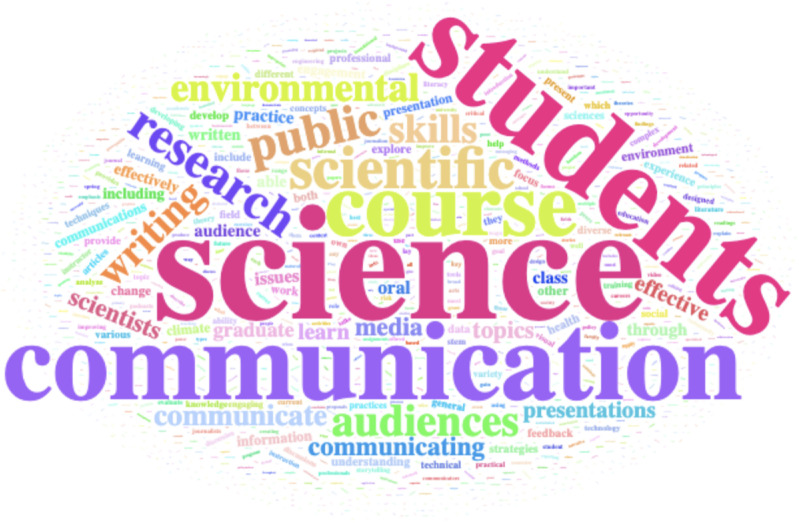
Word cloud of the graduate level science communication course descriptions from the R1 institutions surveyed. Words that appeared more frequently are larger and words that appeared less frequently are smaller.

The descriptions of the courses that met the science communication criteria were analyzed using a frequency-based word cloud (Fig 4). Three words: *science*, *students*, and *communication* have the highest frequency. The next highest frequency words were mainly derivatives of science and communication words such as *scientists*, *scientific*, *communicate*, and *communicating*. The next grouping of words, *research*, *public*, *audiences*, *writing*, *media*, and *presentations*, stresses the important skills and knowledge that are needed for successful science communication. The lower frequency words pertained more towards subject areas such as *environment*, *climate*, *health*, and *social* and are likely a product of the originating course department.

Using a similar approach, graduate level internship courses were discovered with the same methodology as the science communication course analysis. From the search of internship courses, there were so few co-production-based internships that were offered that further analysis was not warranted. Nearly all internships were traditional or offered no description meeting the requirements of analysis and therefore assumed to be a traditional internship course.

### AU NRT climate communication survey results

**Fig 5:**
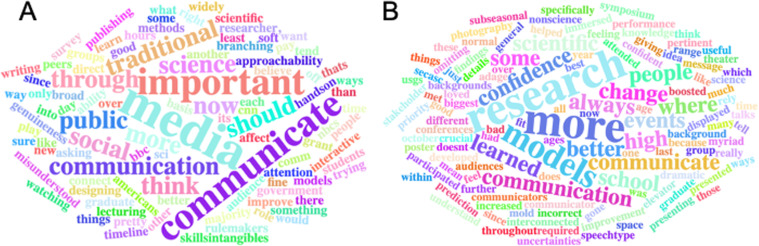
Word clouds of common words used in surveys given to AU NRT students. (A) prior to taking the science communication course and (B) after taking the course.

Open-ended responses to the pre-survey questions (SI Appendix) pertaining to science communication are illustrated in Fig 5A. Results show that among science communication graduate student responders (N = 11), the learning to talk to the *media* and the acknowledgement of the *importance* of science communication were mentioned as reasons taking the science communication course. Other themes represented in the response to prompts that asked about what they hoped to gain from the science communication course included *writing* for non-technical audiences, communicating with *traditional* media vs *social* media, communicating with the *public*, and engaging in *hands-on* activities. Post-survey responses (Fig 5B) show more diversity in the responses focusing on *research*, while the common theme of *communication* is also present. Responses also highlighted the course activities the students received maximum benefits from including a cross-campus climate science *symposium*, the inclusion of guest lecturer from the *theater* departments, elevator *pitch* practice, opportunities for students from various disciplinary backgrounds to *interconnected*, discussions of *stakeholder* needs/interests, and opportunities for students to practice building *confidence*.

### Graduate student internship perspectives

We highlight three different graduate student perspectives (all authors) on the internship process within the AU NRT program. One NRT graduate student completed a non-traditional research internship with a national laboratory researcher. Working hand in hand with a national laboratory researcher was a unique opportunity for the student and helped develop understanding of new concepts and outlooks, as well as learn new skill sets. This student’s internship research was on projections of urban form to understand consequences of future hazards, which fit within their dissertation focus. Though the project idea for the internship was initially identified by the national laboratory researcher prior to the student enrolling in the STEM Studio course, the student was able to link this niche topic into their dissertation research, identified novel ways of approaching the problem, and worked collaboratively to co-create scientific products with the stakeholder. Throughout this process, there was constant communication and resources exchanged using many modes of communication. Although the student had an extensive educational background in civil engineering, jumping into working within the project that included the use of pure statistics and building ideology approaches was still a difficult bridge to cross. The national laboratory researcher was a Human-Dynamics Research Scientist, volunteering their time to guide the student and to aid in the development of proper skills for this project while also sharing insights about their career at the national lab. The team flourished and together, they co-produced a paper presentation at a national conference and are working on a joint journal paper for submission by the end of the student’s degree experience. Exposure to working in conjunction to a National Laboratory has revealed career perspectives in the non-academic research field for the student, as they have been introduced to many other scientists, as well as many different types of research being conducted within the laboratory.

Another NRT student had the opportunity to work with a government entity researching the change of climate risk concerning natural hazards in the US and outlying territories to assess the future risks of these regions. Thus far, this non-traditional research internship has given them insight into working with a diverse team through remote interactions, contractors, and working group collaboration that they otherwise would not have been exposed to inside of the academic research bubble. Through the working group process, the student was on the organizational side of the SDM process for a group of academics, contractors, and various government agencies. Both the student and government researcher valued open communication and invested time and effort to bring everyone to the same level of understanding. The stakeholder of this internship has provided new insights into the research, allowing the developed product to be more impactful and useful for those that will benefit from its findings. The co-produced research will contribute to an online platform assessing climate risks and a journal article. The desired goal of the co-produced research is that people will be able to better assess their current level of climate-associated risk by using this online portal. By understanding how climate risk may change under different climate scenarios, individuals may take action to improve their livelihoods. Through working with this stakeholder, the student will be able to disseminate the results of her research on a larger platform and to a broader audience than it may have received otherwise.

The final perspective details how a NRT graduate student worked on an internship collaboration with a state agency which also was a community stakeholder. This student conducted research involving climate communication with the goal that this research will help to aid vulnerable communities who are experiencing impacts from climate change and do not have the knowledge or resources for adaptation to or mitigation of these impacts. This non-traditional research internship has allowed the student to learn more about the co-production and collaboration process with vulnerable communities. The student learned that in this co-production process, communication is such an important aspect and aids in developing trust with communities. Having honest communication about expectations allows both parties to be on the same page when it comes to how this collaborative process is going to be executed. For example, both the student and the internship mentor have maintained constant communication about timelines for the internship requirements as well as communication with communities about priorities and implementation of research. The collaboration process can be difficult to navigate at times but maintaining accurate and constant communication has proven to be useful from the very beginning. From this internship, the student co-developed the qualitative and quantitative research approaches and tools to be used to collect information from community members, carved out an aspect of the research for inclusion in their dissertation and will be writing manuscripts for academic journals. In addition, they are developing educational materials for vulnerable communities that aim to equip them with increased awareness and knowledge of the impacts of climate change on their community. This internship also provided perspectives about careers outside of academia of interest to the student.

These perspectives highlight the importance and need for graduate students to have exposure to and form networks with non-academic sectors. They provide valuable insights about what students can learn outside the traditional academic setting when provided the structures to conduct not just traditional internships, but ones that are co-produced and that allow them to bring their own creativity and passions to the projects. It also demonstrates that the students can continue to make inroads in their research program, especially when the internships are research based and have been thoughtfully planned out where both stakeholders and student researchers are able to benefit from the research.

Some feedback and recommendations provided from other students in their end of course evaluations were included with the aim of improving the NRT course structures described in this paper. One student stated it would be great if the NRT program offered workshops on science communication in addition to the course. Another student commented that it would be helpful to have more student interaction in the courses and decrease the time devoted to lectures and presentations. Another student suggested that it would be beneficial for the writing assignment in the science communication course to not only include a blog assignment but also a social media post in order to involve a wider range of audiences and communication styles. These valuable recommendations and constructive feedback from the students have aided modifications during additional offerings of the course, which has been taught three additional times since the initial data collected in this paper.

## Discussion

The importance of analyzing the R1 institutions was to find quantitative evidence of possible parallels of similar courses to those offered at AU. From the results, it was shown that the combination of both science communication and structured internship courses is rarely, if at all, offered at R1 universities for graduate students. It is important to incorporate such classes into a graduate student’s education as students may not have exposure to the training of best practices for science communication and working with stakeholders and it is an important part of STEM education and outreach. This lack of training may put graduate students at a disadvantage when sharing their science with lay audiences. It has been shown that science communication surrounding climate change is important for correctly portraying and informing public audiences of the expected impacts [42].

Since AU’s NRT program focused on interdisciplinary solutions towards climate resilience, a science communication course provided a gateway for students to prepare skills to communicate with non-scientists. From the analysis of all R1 institutions, AU is unique in that it offers a science communication (Science Communication) course as well as a structured internship (STEM Studio) course for graduate students. No other university had a similar structure of having both courses formally offered as documented courses on their university course bulletins. This combination of classes allows students to understand how to talk about their specific science in a non-technical way and then use these techniques while completing a non-traditional internship that emphasizes co-production approaches. In the internship, communication techniques are crucial where most information gathered is relayed to a general audience, highlighting the practical applications of the conducted research. In contrast to conventional science internships that offer practical experience, co-production is a collaborative process that involves diverse knowledge sources to address specific problems [21]. Research co-produced with stakeholders, incorporating knowledge transfer, yields more meaningful and accepted outcomes, benefiting all parties involved [3,27].

We want to emphasize the impact that the internship has had on creating a pathway for innovative research and stress the importance of having graduate students collaborate with stakeholders in the real world for perspectives on what research and careers outside of academia look like. Also, students may not have gotten these internships with well-known organizations without the structure of the STEM Studio course and guidance from AU’s NRT program. One of the goals of the NRT program is to develop skills to pursue a variety of STEM careers and encourage partnerships with non-academic agencies such as non-governmental agencies, the private sector, and beyond [43]. While the NRT institutions included in this research may have other ways for their projects to meet these goals, the dual course approach of science communication and STEM Studio for co-production and internships appears to be unique.

This work shows a gap in graduate education across the country with only half of the R1 institutions including a graduate level science communication course that focuses on non-technical or informal audiences, and nearly a third of these institutions offering no graduate level science communication course at all. Further, none of these institutions across the nation offer an graduate level internship course experience outside of the “traditional” internship, where students may not be supported to conduct co-produced work nor work that will easily integrate into their dissertations. This gap indicates that there is a lack of connectivity in supporting graduate students to recognize their audiences, effectively deliver science content to them, and collaboratively work with stakeholders in co-produced research beyond academia.

## Limitations

One limitation in this project is that we searched through individual course catalogs of each R1 institution for current or the previous academic sessions (quarters or semesters). In this analysis, not every university listed their courses in a searchable catalog which made finding courses more difficult. Additionally, only courses offered were considered in our analysis. Institutions could offer workshops or seminars on science communication or co-production that would not have been captured in this study. Another limitation is the sample size of students at AU was limited to a small subset who completed the courses and the surveys available at the point in time of the analysis. Since the AU NRT is an ongoing, externally funded, and niche program, it takes on a limited number of graduate trainees, and thus the sample size is small.

## Conclusion

The call for more effective science communication should incorporate more graduate courses to expose students to both science communication techniques and hands-on experiences with stakeholders. This work was able to showcase the lack of both Science Communication and STEM Studio courses at R1 institutions across the US while illustrating how vital these courses have been to students enrolled in them at AU. These courses have allowed graduate students to gain experiences outside of academia while learning how to effectively communicate the research they are conducting with non-technical audiences. Findings suggest that R1 universities should incorporate such courses into graduate studies to improve learning experiences and train students for a more productive graduate career as well as future career prospects.

### Recommended steps

This perspective paper recommends that nationwide, more universities should offer similarly designed courses to help graduate students develop science communication skills and get hands-on experience of collaborating with stakeholders and practitioners. Also, rather than being a strictly classroom endeavor, institutions should consider engaging campus resources and career services to aid in establishing strong connections between stakeholders and graduate students that are more than a traditional internship. These internships should include co-produced research elements to develop future leaders that are able to apply their research to real-world problems and provide stakeholders useful products in actionable science.

Our recommendation is that students should be supported to have the option to work with stakeholders in a research field they are interested in pursuing. While all universities may not have the opportunity to have a NRT or similar funded program, all universities can incorporate co-production and knowledge transfer between graduate students and stakeholders, thus providing better skills for students to obtain suitable job opportunities in the future. One way to implement this recommendation is to have the university career service offices or university outreach offices help with the organization of internships with stakeholders for graduate students. Another way is maintaining an embedded structure through a formal or informal course that guides and supports students as they complete their internships, maximizing opportunities to include the work in their dissertation whenever possible.

A final recommendation is that a science communication course aimed at non-technical audiences should be included in graduate student coursework. Science communication can be used in various types of settings from on and off-campus outreach events, social media, and talking to a family member or friends about a science topic. If one department hosts a science communication course, then it should be open to more disciplines than just to those students housed in that department. This approach will benefit the department offering the course by having more students enrolled, benefit students across disciplines, and create a more robust course with multi-disciplinary perspectives.

The significance of this work highlights that AU’s NRT program through two niche structured courses on graduate students is a novel program and very few other institutions and departments currently provide this combination of courses. These findings highlight that there are national gaps in this approach, and we encourage more US institutions to adopt similar innovative approaches to support graduate students in STEM. Future research could expand on this study by broadening the survey participants to a larger, more diverse population that ranges over multiple universities.

## Supporting information

S1 Appendix The science communication questions were asked of NRT trainees at AU. The drop-down values for the second column (importance) were as follows: “extremely important,” “very important,” “important,” “somewhat important,” and “not important.” The drop-down values for the third column (confident) were: “not at all confident,” “somewhat confident,” “confident,” “very confident,” and extremely confident.”.
(PDF)
