# Supplementary material for: Benefits of a non-traditional science communication and internship experience based on research from the National Science Foundation Research Traineeship at a Research Intensive University
Source: PLoS One. 2025 Apr 22;20(4):e0320372. doi: 10.1371/journal.pone.0320372 (PMC12013938; doi:10.1371/journal.pone.0320372)
Supplement: S1 Appendix — The drop-down values for the second column (importance) were as follows: “extremely important,” “very important,” “important,” “somewhat important,” and “not important.” The drop-down values for the third column (confident) were: “not at all confident,” “somewhat confident,” “confident,” “very confident,” and extremely confident.”. (PDF) [file pone.0320372.s001.pdf]

Science Communication: For each item, **please select the response option that is most appropriate** regarding what skills, knowledge, or activities you think are important for you to learn during graduate school and the amount of confidence you have with that area.

	To what degree do you think this area is important for you to learn about as you go into your STEM career?	How confident do you feel in effectively performing the stated skill?
Communicating with other scientist in my field		
Communicating with other scientists outside of my field		
Communicating with stakeholder groups about my research		
Communicating with the public about my research		
Communicating about my research on social media		
Communicating with my friends and family about my research		
Presenting a scientific talk		
Presenting a short "elevator" speech		
Communicating with the public about how science is done		
Communicating with the public about variability and uncertainty		
Communicating with the public about climate models (e.g., computer, physical or conceptual models)		

Please write any additional skills you think are important for your learning related to **Science Communication**. Please also provide any comments or questions related to your responses in the section above.
